# Diseases of the Genitourinary Organs and the Kidney

**Published:** 1908-03

**Authors:** 


**Diseases of the Genitourinary Organs and the Kidney.** By **Robert H. Greene,** M.D., Professor of Genitourinary Surgery at the Fordham University, New York; and **Harlow Brooks,** M.D., Assistant Professor of Pathology, University and Bellevue Hospital Medical School. Octavo of 536 pages, illustrated. Philadelphia and London: W. B. Saunders Company, 1907. (Cloth, $5.00; half morocco, $6.50 net prices.)

If there is one branch of medicine which needs help in a literary way, it is genitourinary work. There are few volumes in the mass of treatises, textbooks, systems and reprints which approach the point where criticism may cease and honest commendation begin. There is either too much or too little; too much opinion or none at all; there is hardly a work which may be considered to approach the happy medium.

There was, it must be confessed, a flicker of hope, when the announcement of the forthcoming publication of Green and Brooks's book was made, that now would come a literary Moses to lead the bewildered general practitioner and the hungry student out of the wilderness. The book has made its appearance and it is disappointing. The more important diseases of the genito-urinary tract are discussed, but the discussion will do little more than start some over eager student into a field of fads that can do him little good. It must not be thought by this that there is no good to be found in the book; there is much that is excellent, and almost brilliant are those portions which deal with pathology; yet the few bright spots are dimmed by the glare of error which strikes one forcibly even in a casual reading.

It is only necessary to call attention to the woeful chapter on gonorrhea wherein it is advised to withhold all local treatment until at least the fourth week of the disease,—a treatment which is not in accord with modern methods by any manner of means. The authors' abortive treatment for gonorrhea is difficult of serious consideration, but it is a good thing to read in order to know what not to do. As a whole the book lacks that satisfying tone which a really perfect work should possess. It is more or less superficial; and as for errors, they pop up like mushrooms on a misty morning. A thorough editing, a careful revision and a new edition would do much toward making a better book of it.

